# Prevalence, associated factors, and functional impact of low back pain among pregnant women attending antenatal care in Ouagadougou, Burkina Faso: A cross-sectional study

**DOI:** 10.1371/journal.pone.0360908

**Published:** 2026-10-23

**Authors:** Mariam Ouédraogo, Marie Banyama Diabri, Yannick Laurent Tchenadoyo Bayala, Issa Ouédraogo, Enselme Zongo, Aboubakar Ouédraogo, Fulgence Kaboré, Wendlassida Joelle Stephanie Zabsonre-Tiendrebeogo, Dieu-Donné Ouédraogo

**Affiliations:** 1 Department of Rheumatology, Bogodogo University Teaching Hospital, Ouagadougou, Burkina Faso; 2 Department of Rheumatology, Health Sciences Training and Research Unit, Joseph Ki-Zerbo University, Ouagadougou, Burkina Faso; University of Uyo, NIGERIA

## Abstract

**Background:**

Low back pain (LBP) is a common musculoskeletal disorder during pregnancy that can affect quality of life. While extensively studied in high-income countries, data from sub-Saharan Africa remain scarce. This study aimed to determine the prevalence, identify associated factors, and assess the impact of LBP among pregnant women in Ouagadougou, Burkina Faso.

**Methods:**

A cross-sectional study was conducted from February to May 2024 in 11 primary healthcare centers across five health districts of Ouagadougou. Pregnant women receiving antenatal care were consecutively recruited. Data, including sociodemographic characteristics, obstetric history, and the Pregnancy Mobility Index (PMI), were collected via standardized questionnaires. Multivariable logistic regression was performed to identify factors associated with LBP.

**Results:**

Among the 1,159 pregnant women (mean age 26.86 ± 5.91 years), the prevalence of LBP was 21.83%. The mean PMI was 13.31 ± 9.40. Although 45.42% of women reported reduced activity levels, 71.71% considered their LBP to be nonsignificant. Multivariable analysis identified urban residence (aOR: 1.85, 95% CI: 1.20–2.93, p = 0.007), low activity level (aOR: 1.57, 95% CI: 1.14–2.15, p = 0.006), and personal history of low back pain (aOR: 14.89, 95% CI: 9.64–23.53, p < 0.001) as associated factors for LBP.

**Conclusion:**

Although LBP affects one in five pregnant women in Ouagadougou, it is often considered nonsignificant despite its impact on daily activities. Urban residence, reduced activity level, and personal history of low back pain emerged as key associated factors.

## 1. Introduction

Low back pain is defined as localized pain between the costal margin and the lower gluteal fold, with or without irradiation of the lower limbs [1]. Pregnancy results in many physiological changes, including increased weight and tissue elasticity. These physiological changes affect lumbar spine mechanics and load on osteoarticular structures, as they lead to different functions of the lumbar spine and stresses on osteoarticular structures [2].

Low back pain (LBP) is a common musculoskeletal problem during pregnancy [3]. According to a review by Wu et al. covering studies published between 1982 and 2002, the reported prevalence of pregnancy-related low back pain ranged widely, from 3.9% to 89.9%, with a median of 49.0% [4]. In 2023, Salari et al. conducted a meta-analysis of 28 studies worldwide, reporting an overall prevalence of low back pain of 40.5% during pregnancy [5]. According to a study conducted in Turkey in 2018 involving a population of 1,500 pregnant women, the prevalence of low back pain was 53.93% [6]. The differences observed are undoubtedly due to the sociodemographic and health determinants specific to each country and to the study methodology used.

To our knowledge, few studies on this subject have been carried out in sub-Saharan Africa [7–10]. In black African series, the prevalence of low back pain ranged from 12.4% to 62%; the study population sizes were 303 in South Africa, 404 in Malawi and 478 in Nigeria [7–10]. In the Nigerian study by Omoké et al., the onset of pain was predominantly in the third trimester, and the mean pain intensity was 4.3 ± 1.36. Logistic regression analysis revealed that LBP during a previous pregnancy (aOR: 24.76, 95% CI 6.88–89.11; p < 0.001), macrosomia (aOR: 4.15 (95% CI 2.05–8.42); p < 0.001) and the absence of domestic help (aOR: 0.50 (95% CI 0.31–0.82); p = 0.006) were independent associated factors for LBP during pregnancy among women [7]. This pain can lead to incapacity or disability, resulting in a poor quality of life. In Indonesia, a study reported that pregnant women suffering from back pain had a lower quality of life than those who did not [11].

Rheumatology in Burkina is characterized by an increasingly frequent demand for the management of low back pain in pregnant women. This led to the following research questions: What is the prevalence of low back pain in pregnant women? What is its impact on pregnant women? The aim of this study was to assess the prevalence, determine the associated factors and impact of low back pain in pregnant women in the city of Ouagadougou in 2024. Given the scarcity of data on this condition in sub-Saharan Africa, the findings of this study are expected to inform the integration of routine musculoskeletal screening into antenatal care protocols in Burkina Faso and comparable settings, and to support the development of context-appropriate clinical guidance and public health strategies addressing pregnancy-related low back pain.

### 2. Patients and method

**Fig 1:**
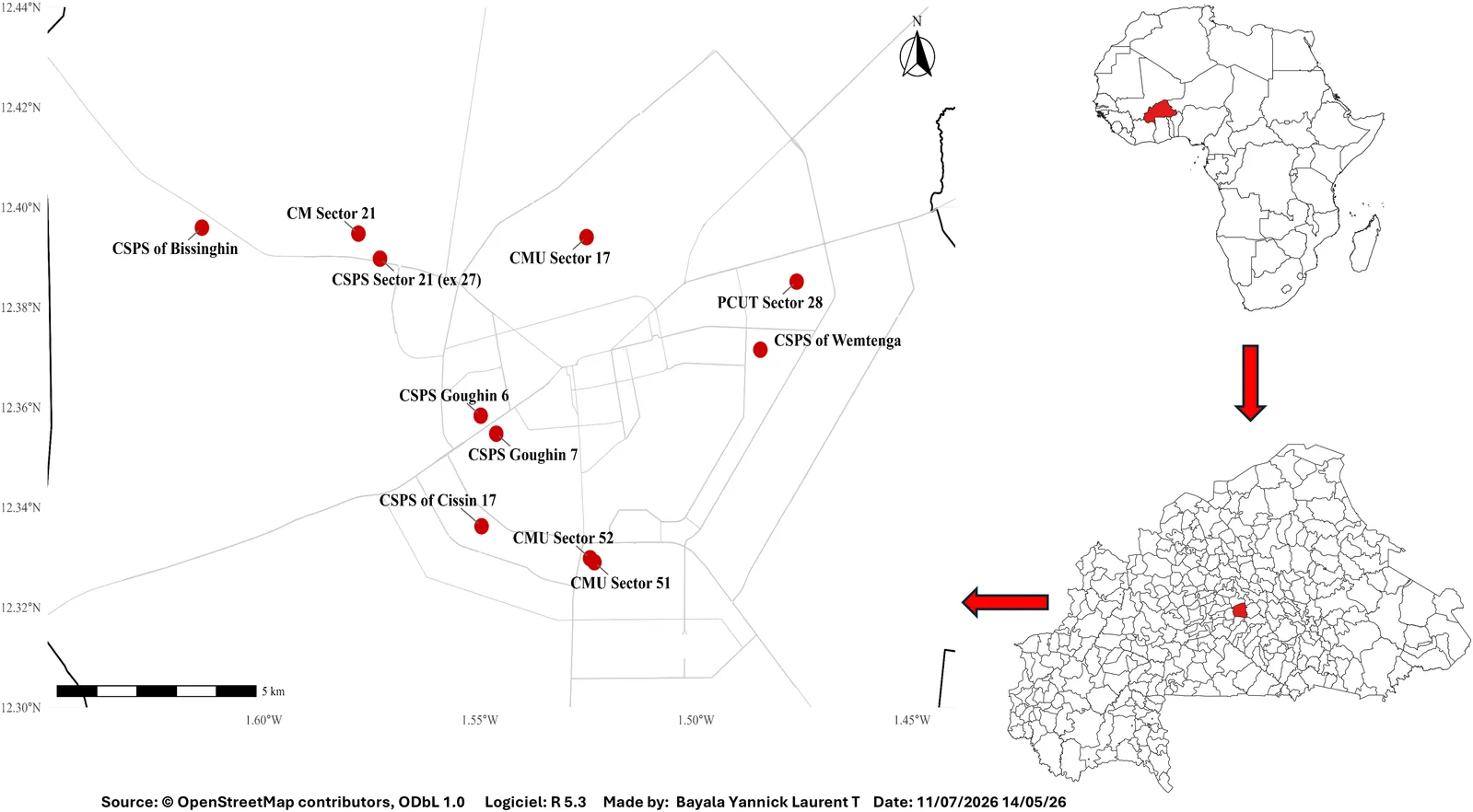
Geographic distribution of the 11 participating health centers across Ouagadougou, Burkina Faso. Red dots indicate the location of each health and social promotion center where data collection took place between February 19 and May 03, 2024. The city boundary corresponds to the administrative limits of the Ouagadougou department, Kadiogo province, Centre region, Burkina Faso. CMU: Urban Medical Center (Centre Médical Urbain); CSPS: Health and Social Promotion Center (Centre de Santé et de Promotion Sociale); CM: Medical Center (Centre Médical); PCUT: Emergency Consultation and Treatment Post (Poste de Consultation et de Traitement d’Urgence). Map created by the authors using R. Road network: Contains information from OpenStreetMap and OpenStreetMap Foundation, which is made available under the Open Database License. Country and administrative boundaries (inset maps): Natural Earth (public domain)..

### 2.1 Research site

Our research was conducted in Ouagadougou, the capital of Burkina Faso (Fig 1). It is a landlocked country in west Africa with 20,505,155 inhabitants; the population of women of childbearing age (15–49 years) was 4,949,953; the Ouagadougou women population was estimated to be 1,231,709 in 2019; and the total fertility rate in Ouagadougou is 4.0 children per woman [12].

The healthcare system in Burkina Faso is structured into three administrative levels:

- the central level, comprising the central structures attached to and organized around the minister’s office and the general secretariat;
- the intermediate level comprises the 13 regional health directorates and hospitals;
- the peripheral level comprises 70 health districts, the most decentralized operational units of the national health system, structured around health and social promotion centers (CSPS) and medical centers with surgical annex (CMA) [13].

### 2.2 Types of research

This descriptive and analytical cross-sectional study was conducted from February to May 2024 in 11 health centers of the city of Ouagadougou (Fig 1).

### 2.3 Study population

Pregnant women who were undergoing a prenatal consultation in health center were included. Health centers were purposively selected based on antenatal consultation volume: the health and social promotion centers with the highest number of prenatal consultations, based on routine attendance records reported by the five health districts prior to the study period, were selected for inclusion. A total of 11 health and social promotion centers were included in the study, and data were collected over a 2-week period at each center.

#### Inclusion criteria.

Pregnant women who received prenatal care in health districts and for whom written consent had been obtained were included.

#### Exclusion criteria.

Pregnant women with a history of lumbar spine trauma, lumbar spine surgery, pelvic trauma or pelvic surgery were excluded from the study.

### 2.4 Sampling

An a priori minimum sample size of 380 was calculated using the Schwartz formula (Z = 1.96, 95% confidence, expected prevalence = 45%, δ = 0.05), for reference purposes. However, this was a facility-based study using a purposive, exhaustive recruitment strategy: all eligible women presenting for antenatal consultation at each of the 11 selected health centers were enrolled over a fixed two-week period per center, consistent with common practice in hospital- and facility-based studies. This approach resulted in a final sample of 1,159 pregnant women, exceeding the calculated minimum and providing additional precision for subgroup and multivariable analyses.

### 2.5 Data collection

The data was collected with questionnaires developed on the KoBo ToolBox and implemented with smartphones. In each health center, each team was equipped with examination equipment, consent forms, pregnancy mobility index forms and a smartphone containing the KoBo ToolBox collection form. Each team was set up in an equipped room. All pregnant women who came for prenatal consultation were subjected to the questionnaire. Initially, the purpose and conditions of the study were explained to each participant. She was free to give or withhold written informed consent. Recruitment aimed to include all eligible women presenting for antenatal consultation at each center during the data collection period. Second, they were interviewed, and those with complaints were examined by the investigators. When those with complaints were examined, a diagram delimiting the pelvic girdle was presented to each participant for greater precision in the answers. The patient’s privacy was respected during the examination. The data collection team consisted of supervisors and interviewers. The supervisors were rheumatologists, seniors, statisticians and epidemiologists. The interviewers were rheumatology residents. All interviewers received standardized training prior to data collection, covering questionnaire administration, the pelvic girdle diagram used to enhance response precision, and clinical examination procedures, to ensure consistency across the 11 participating health centers. Participant recruitment was conducted from February 19 to May 03, 2024.

### 2.6 Research variables

The dependent variable was low back pain, defined as localized pain between the costal margin and the lower gluteal fold, with or without irradiation of the lower limbs. It was measured by self-report during the standardized interview, as pain experienced at any point since the beginning of the current pregnancy (period prevalence). Pain intensity was not measured, and recurrent versus chronic pain patterns were not differentiated.

Independent variables were measured as follows. Sociodemographic variables included age (in completed years, self-reported), residence (urban or rural, based on the participant’s declared place of residence), occupation (self-reported and categorized into retailer, housewife, civil servant, student, or other), marital status (self-reported, categorized as in couple [married or cohabiting] or not in couple [single or widowed]), educational level (self-reported highest level attained: no formal education, primary, secondary, superior, or French-Arabic/vocational school), and presence of home help (yes/no). Personal history of low back pain was self-reported as having experienced low back pain during a previous pregnancy (yes/no). Physical measures included weight (kg) and height (cm), measured using a standard scale and stadiometer; body mass index (kg/m^2^), calculated from measured weight and height; uterine height (fundal height) (cm), measured by the examining clinician using a standard obstetric tape measure; gravidity and parity (self-reported, verified against antenatal records where available); intergenesis interval (years since the most recent prior pregnancy, self-reported); and gestational age (weeks), calculated from the reported date of last menstrual period or ultrasound dating where available. The Pregnancy Mobility Index (PMI), a validated self-report instrument assessing mobility related to back and pelvic girdle pain during pregnancy, was administered to all pregnant women reporting low back pain. This study followed the original item-level response format of the PMI (each item scored 0–3); however, the total score was calculated as the raw sum of item scores, without the standardized 0–100 transformation used in the original instrument. As such, established severity thresholds derived from the transformed 0–100 scale do not directly apply to the raw scores reported here. Activity level during pregnancy was self-reported in comparison with activity before pregnancy and classified as low (lower than before pregnancy), normal (similar to before pregnancy), or intense (greater than before pregnancy).

### 2.7 Analysis and processing of data

STATA software was used for data analysis. The chi-square test was used to search for associations. Quantitative variables are expressed as the means ± standard deviations or as medians and quartiles, according to the distribution curve of the values of these variables (symmetrical or not). Qualitative variables are expressed as percentages and headcounts. All variables associated with low back pain at p ≤ 0.20 in univariate analysis were entered into an initial multivariable logistic regression model. A backward elimination procedure was then applied automatically in Stata, sequentially removing the variable with the highest p-value above the 0.05 threshold until only variables with p ≤ 0.05 remained in the final model. This approach yielded the adjusted odds ratios (aOR) presented as the final model. Multicollinearity among variables retained in the final multivariable model was assessed using variance inflation factors (VIF).

Missing data patterns were assessed for all variables included in the analysis. For multi-select checkbox items collected via the KoBoToolbox platform, unselected checkbox values were recorded as missing (NA) by the data collection software when the corresponding question group was answered but a specific item was not selected; these values were therefore recoded as absent (0) rather than treated as missing data, consistent with the skip-logic structure of the electronic questionnaire. This recoding was validated by confirming that resulting variable distributions matched the frequencies reported in descriptive tables. For variables subject to genuine missingness independent of questionnaire skip logic, namely newborn birth weight and the Pregnancy Mobility Index score, which was administered only to women reporting low back pain a complete-case approach was used, and these variables were analyzed only among participants with available data, as reported in the corresponding tables. No imputation was performed for genuinely missing values.

### 2.8 Ethical considerations

The protocol was reviewed and approved by the Ethics Committee for Health Research (Deliberation No 2023-12-284). We also obtained authorization from the Ministry of Health to conduct the study (reference No 2024/0415/MSHP/SG/DGESS of February 1, 2024). The study adhered to the ethical principles of the Helsinki Declaration.

Beyond these approvals, ethical principles were applied as follows. Prior to data collection, the purpose, procedures, risks, and voluntary nature of participation were explained individually to each woman in a private setting, respecting her autonomy to accept or decline participation without consequence for her antenatal care. Written informed consent was obtained directly from each participant prior to any data collection or clinical examination. This included 14 pregnant minors (aged 15–17 years, 1.21% of the sample), for whom the approved protocol explicitly authorized autonomous informed consent without parental or guardian involvement, consistent with recognized ethical provisions granting pregnant minors autonomy over decisions related to their reproductive and maternal health care. No distinct assent procedure was used, as the approved protocol considered full informed consent from the minor herself to be both necessary and sufficient in this context. Confidentiality was maintained throughout data collection and clinical examination, which was conducted in a private room, and participant data were anonymized prior to analysis.

## 3. Results

### 3.1 Characteristics of the pregnant women

One thousand one hundred and fifty-nine pregnant women were included during the study period (Fig 2). The mean age was 26.86 years ± 5.91 years, with extremes of 15 years and 46 years. Table 1 shows the sociodemographic characteristics of the pregnant women.

**Table 1:** Distribution of 1159 pregnant women according to socio-demographic characteristics (Ouagadougou from February 19 to May 03, 2024).

Variables	Frequency	Percentage
**Age (years), mean ± SD**	26.86 ± 5.91	
**Place of residence**		
Rural	213	18.38
Urban	946	81.62
**Socio-professional status**		
Retailer	475	40.98
Housewives	405	34.94
Civil servant	55	4.76
Student	101	8.71
Other *	123	10.61
**Marital status**		
Cohabiting	237	20.45
Single	72	6.21
Married	849	73.25
Widow	1	0.09
**Educational level**		
No formal education	284	24.50
Primary	262	22.61
Secondary	469	40.47
Superior	120	10.35
Other (french-arab school / vocational school)	24	2.07
**Activity level**		
Low	421	36.33
Intense	21	1.81
Normal	717	61.86
**Home help**		
No	717	61.86
Yes	442	38.14

Other professions include*: cleaning staff, weaver, restorer, cashier, domestic helper, gardener, storekeeper, traditional healer, waitress, intern, hotel worker, medical delegate, freight forwarder, accountant, facilitator, customer advisor, farmer, kiosk manager, cook, assembly technician, telecom manager, entrepreneur.

**Fig 2:**
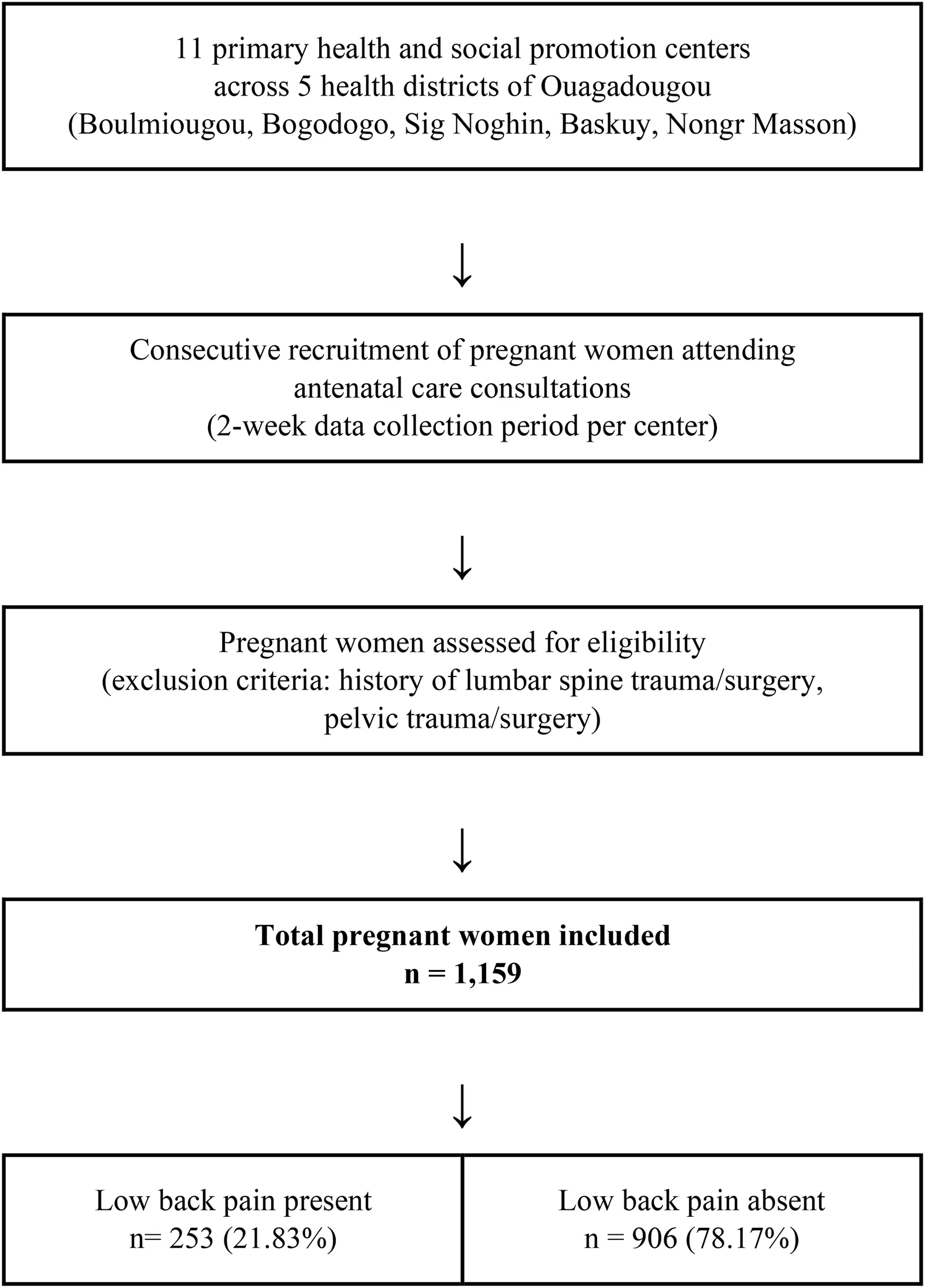
Flow diagram of participant recruitment. Note: The number of women approached and the number of refusals during recruitment were not systematically recorded; therefore, a formal response rate could not be calculated. This diagram reflects the flow from center-level recruitment to final analytic inclusion.

Table 2 shows the clinical characteristics of the pregnant women and their pregnancies. Two hundred and fifty-three pregnant women (21.83%, 95% CI: 19.50–24.34%) had low back pain.

**Table 2:** Distribution of 1159 pregnant women by clinical characteristics (Ouagadougou. February 19 to May 03. 2024).

Variables	Mean ± SD	Extremes
Weight (kg)	68.47 **±** 12.85	40 and 137
Size (cm)	163.09 ± 6.09	150 and 200
Body mass index (BMI) (kg/m²)	25.76 ± 4.65	15.62 and 57.45
Parity	1.22 ± 1.52	0 and 14
Gravidity	2.35 ± 1.53	1and 12
Uterine height (fundal height) (cm)	21.68 ± 8.93	0 (globular) and 40
Interpregnancy interval	2.22 **±** 2.42	0 and 15
Gestational age (weeks of gestation)	25.26 **± 7.72**	4 and 42
	**Frequency**	**Percentage**
**Gestational age (trimester)** 1^st^ trimester	242	20.88
2nd trimester	415	35.81
3^rd^ trimester	502	43.31
**Personal history of low back pain** No	1038	89.56
Yes	121	10.44

### 3.2 Functional impact: Mobility, activity level, and self-perceived severity among women with low back pain

Functional impact was assessed among the 251 women reporting low back pain using the Pregnancy Mobility Index, self-reported activity level, and self-perceived severity of the condition. The mean pregnancy mobility index was 13.31, and 71.71% of pregnant women with low back pain felt that their condition was not significant. One hundred and fourteen pregnant women (45.42%) had a low level of activity. Table 3 shows the distribution of the 251 pregnant women according to these functional impact indicators.

**Table 3:** Distribution of the 251 pregnant women with low back pain according to functional impact indicators (Pregnancy Mobility Index score, activity level, and self-perceived severity), Ouagadougou, February 19 to May 03, 2024.

Variables			Frequency	Percentage
	Mean + /- SD*	Extreme		
**Pregnancy Mobility Index**	13.31 + /- 9.40	1-63		
**Activity level**	Low		114	45.42
	Intense		7	2.79
	Normal		130	51.79
**Personal assessment of low back pain**	Significant problem		71	28.29
	Insignificant problem		180	1.1

*: standard deviation. Note: This table presents data among the subgroup of women reporting low back pain (n = 251), as these measures are specific to symptomatic participants.

### 3.3 Associated factors

#### Univariate analysis.

Univariate analysis revealed that increased uterine height (p < 0.001), increased gestational age (p = 0.004), increased gravidity (p = 0.004), and shorter interpregnancy intervals (p = 0.002) were significantly associated with low back pain (LBP). Women with a higher BMI (p = 0.105) had a borderline association with LBP. Socioprofessional status (p = 0.004), education level (p = 0.024), and low activity level (p = 0.001) were also significant factors. A history of LBP was strongly associated with current LBP (p < 0.001). No significant associations were found with marital status (p = 0.264), home help (p = 0.530), or twin pregnancies (p = 0.624). Tables 4 and 5 show the results of the univariate analyses between low back pain and the independent variables.

**Table 4:** Comparison of quantitative characteristics between pregnant women with and without low back pain (Ouagadougou, February 19 to May 03, 2024).

Variable mean	Low back pain	No low back pain	*p* value
Age (years)	26.22	27.04	0.42
Weight (kg)	70.25	67.98	0.013
Size (cm)	163.67	162.93	0.088
BMI (kg/m2)	26.18	25.64	0.105
Uterine height (cm)	25.05	20.74	0.000
Gestational age (weeks of gestation)	26.50	24.92	0.004
Gravidity	2.60	2.28	0.004
Parity	1.39	1.17	0.040
Interpregnancy interval (year)	2.63	2.10	0.002

**Table 5:** Univariate analysis. Association between sociodemographic characteristics and low back pain in pregnant women (Ouagadougou from February 19 to May 03. 2024).

Variable	OR	95% CI	*p*-value
Socio-professional status			
Civil servant (ref)	1.00	—	—
Other	0.77	0.25–2.86	0.667
Retailer	0.69	0.22–2.57	0.538
Student	1.07	0.30–4.39	0.918
Housewife	0.59	0.19–2.18	0.378
Marital status			
Not in a couple (ref)	1.00	—	—
In a couple	1.62	0.87–3.29	0.152
Educational level			
Higher education (ref)	1.00	—	—
Other (French-Arabic/vocational school)	1.18	0.42–3.03	0.735
No formal education	0.54	0.32–0.91	0.020
Primary	0.95	0.58–1.57	0.830
Secondary	0.83	0.53–1.33	0.425
Activity level			
Normal (ref)	1.00	—	—
Low	1.61	1.21–2.14	0.001
Intense	2.20	0.82–5.39	0.096
Home help			
No (ref)	1.00	—	—
Yes	1.15	0.86–1.52	0.340
Personal history of low back pain			
No (ref)	1.00	—	—
Yes	14.82	9.67–23.24	<0.001
Gestational age (trimester)			
Third trimester (ref)	1.00	—	—
First trimester	0.95	0.58–1.50	0.829
Second trimester	1.19	0.88–1.60	0.254
Twin pregnancy			
No/unknown (ref)	1.00	—	—
Yes	3.60	0.43–30.14	0.201

**Note:** OR, crude odds ratio; CI, confidence interval; ref, reference category. Low back pain was defined as self-reported pain experienced since the onset of the current pregnancy.

#### Multivariable analysis.

Candidate variables associated with LBP at p ≤ 0.20 in univariate analysis (residence, educational level, activity level, marital status, and personal history of LBP) were entered into an initial multivariable model. Automatic backward elimination retained three variables in the final model: residence, activity level, and personal history of LBP (Table 6). Educational level and marital status did not survive the elimination procedure. Gestational age and uterine height, despite their univariate associations with LBP (Table 4), were not retained in the final multivariable model.

**Table 6:** Multivariable analysis association between characteristics of pregnant women and low back pain (Ouagadougou, February 19 to May 03, 2024).

Variable	aOR	CI 95%	p-value
Residence			
Rural (ref)	1.00	—	—
Urban	1.85	1.20–2.93	0.007
Activity level			
Normal (ref)	1.00	—	—
Low	1.57	1.14–2.15	0.006
Intense	1.16	0.36–3.37	0.800
Personal history of low back pain			
No (ref)	1.00	—	—
Yes	14.89	9.64–23.53	<0.001

*Note: Variables were selected using univariate screening (p ≤ 0.20) followed by automatic backward elimination (retention threshold p ≤ 0.05). Gestational age and uterine height were not retained. aOR: adjusted odds ratio; CI: confidence interval; ref: reference category.*

Women living in urban areas had higher odds of LBP than those in rural areas (aOR: 1.85, 95% CI: 1.20–2.93, p = 0.007). Low activity level was associated with higher odds of LBP compared with normal activity (aOR: 1.57, 95% CI: 1.14–2.15, p = 0.006), while intense activity showed no significant association (aOR: 1.16, 95% CI: 0.36–3.37, p = 0.800). Personal history of low back pain showed the strongest association, with nearly a 15-fold increase in odds among women reporting a prior episode (aOR: 14.89, 95% CI: 9.64–23.53, p < 0.001).

The final model showed a good fit, with a non-significant Hosmer-Lemeshow test (χ^2^ = 0.42, df = 3, p = 0.936). Model discrimination was acceptable, with an area under the ROC curve of 0.699 (95% CI: 0.662–0.737) (Fig 3). McFadden’s pseudo-R^2^ was 0.152, indicating that the model explained a moderate proportion of the variability in LBP occurrence. Variance inflation factors for all retained variables were close to 1 (range: 1.02–1.03), indicating no multicollinearity.

**Fig 3:**
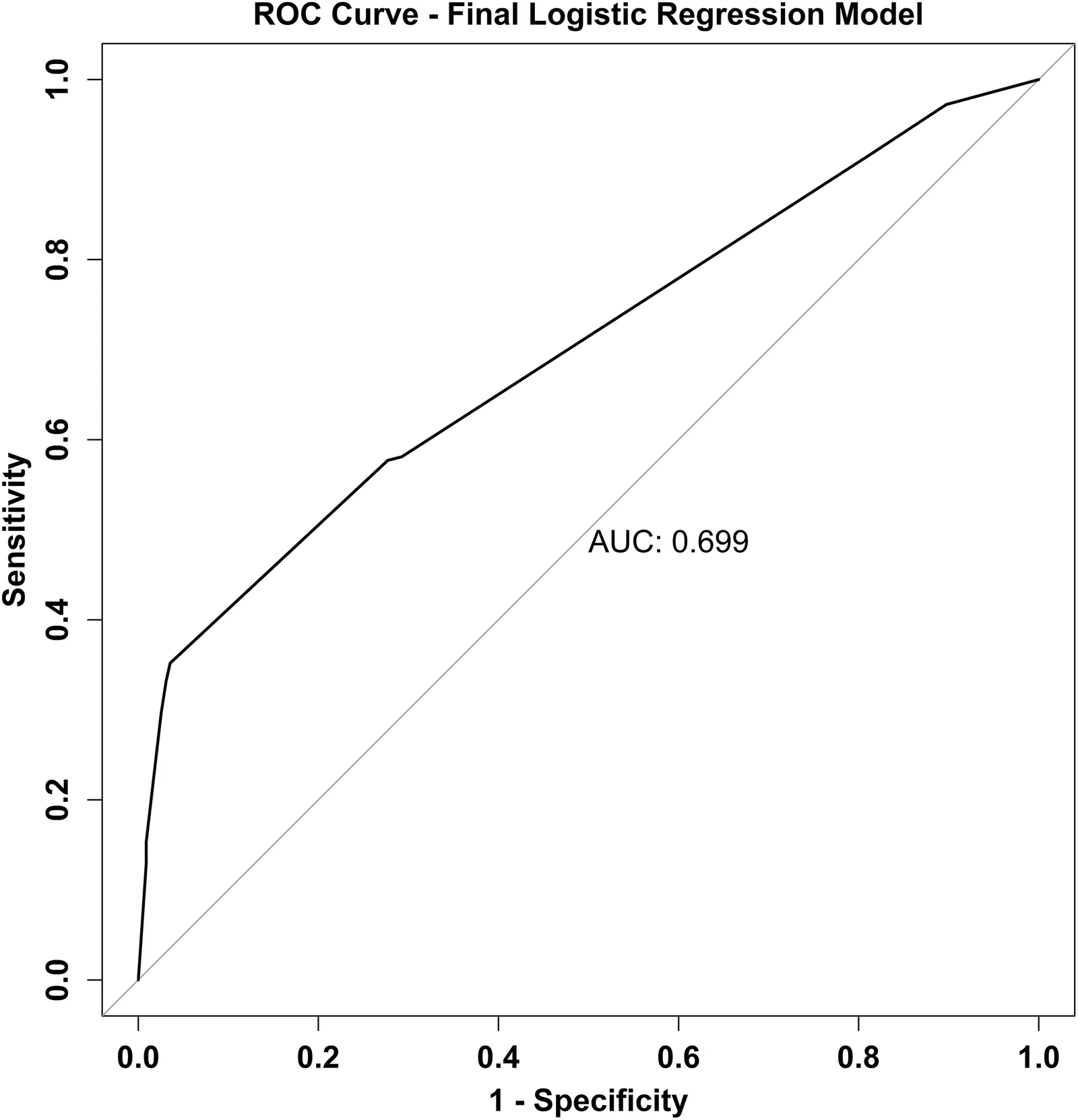
Receiver operating characteristic (ROC) curve for the final multivariable logistic regression model predicting low back pain among pregnant women. The model includes urban residence, activity level, and personal history of low back pain as predictors. The area under the curve (AUC) was 0.699 (95% CI: 0.662–0.737), indicating acceptable discriminatory ability.

## Discussion

The prevalence of low back pain in pregnant women in our series was 21.83%. This prevalence varies from 12.4% to 60% in African studies [7–10]. In the series by Omoke et al. in Nigeria, of the 478 women interviewed, 138 (28.9%) reported LBP in the index pregnancy [7]. In a retrospective cohort study, among 167,396 pregnancies among South Carolina Medicaid beneficiaries, the overall prevalence of low back pain was 15.6% [14]. In a review of 28 studies with a sample size of 12,908 people, the global prevalence of back pain was 40.5% during pregnancy [5]. Pain is subjective. The reported differences in the prevalence of low back pain in women may be due not only to the sociocultural context but also to methodological differences. Indeed, the prevalence of LBP varies according to the trimester of pregnancy [5]. Additionally, according to a meta-analysis, the global prevalence of back pain in the first trimester of pregnancy is 28.3, that in the second trimester is 36.8, and that in the third trimester of pregnancy is reported to be 47.8 [5]. Backhausen et al reported that the overall prevalence of low back pain was 76% at 20 weeks and 90% at 32 weeks of gestation [15]. In our series, the crude proportion of low back pain did not increase progressively across trimesters (Table 5), in contrast with most studies reporting a progressive increase [5,7,9,10,14]. Gestational age was not retained as an associated factor in the final multivariable model after adjustment for residence, activity level, and personal history of LBP, suggesting that its univariate association with LBP may be explained, at least in part, by its correlation with these retained variables.

Several methodological factors may account for this lower prevalence. First, our study relied on a single-point self-report of current pain without a standardized recall period, which may have led to underestimation compared with studies using a defined recall window. Second, facility-based recruitment at antenatal care visits may have excluded women with more severe symptoms who were unable to attend, or who sought care elsewhere. Third, cultural normalization of musculoskeletal discomfort during pregnancy, as reflected in the finding that 71.71% of affected women rated their pain as insignificant, may have led to systematic underreporting. Finally, differences in operational case definitions across studies, discussed further below, limit direct comparability of prevalence estimates.

The mean pregnancy mobility index was normal in our series. One hundred and fourteen pregnant women with low back pain (45.42%) had a low level of activity, and despite this, 71.71% felt that their health status was not significant. A cross-sectional study involving the successive recruitment of pregnant women at two district hospitals in Nepal reported low disability despite severe pain intensity and poor beliefs in recovery after delivery [16]. This discrepancy between functional limitation and self-perceived severity may reflect several interrelated factors. Musculoskeletal discomfort during pregnancy may be culturally normalized and perceived as an expected, temporary consequence of pregnancy rather than a condition warranting medical attention, particularly in settings where maternal health priorities have historically centered on infectious and obstetric complications rather than musculoskeletal morbidity. This normalization may be compounded by limited awareness of available management options and by competing daily demands that leave little room to prioritize symptom reporting. Similar patterns of underreporting despite functional impairment have been described in other low-esource settings [16], suggesting that self-perceived severity may substantially underestimate the true burden of pregnancy-related low back pain in similar contexts. These findings underscore the importance of proactive clinical screening for low back pain during antenatal visits, rather than relying solely on spontaneous patient reporting, and highlight an opportunity to strengthen antenatal counseling around musculoskeletal symptoms as a routine component of maternal care.

The condition of pregnant women with LBP is presumably influenced by a variety of factors [15]. In our final model, urban residence, low activity level, and personal history of low back pain emerged as the associated factors, while educational level, gestational age, and uterine height, though associated with LBP in univariate analysis, did not remain significant after adjustment.

The association between urban residence and LBP (aOR: 1.85) has not been consistently reported in prior studies and warrants cautious interpretation. Plausible explanations include differences in occupational patterns, with urban women more frequently engaged in sedentary work or prolonged standing in retail or service occupations, as well as differential access to and use of antenatal care services, which may result in more complete symptom reporting among urban participants compared with their rural counterparts.

Low activity level was associated with higher, rather than lower, odds of LBP in this final model (aOR: 1.57), consistent with the univariate findings and with the biological plausibility that pain leads women to reduce their activity. However, because activity level and LBP were measured concurrently in this cross-sectional design, reverse causation remains a plausible explanation: women experiencing pain may have reduced their activity level in response to the pain itself, rather than reduced activity contributing causally to its onset. This finding should therefore be interpreted as an association without inferring a specific causal direction. Caputo et al. reported that working more than 8 hours per day and routinely lifting heavy objects at work increased the odds of LBP [17], supporting the broader role of activity-related mechanical load in this condition.

Personal history of low back pain showed by far the strongest association with current LBP, both in univariate analysis (71.9% among women with prior history versus 15.8% among those without) and after adjustment (aOR: 14.89). This finding is consistent with prior literature identifying previous LBP as one of the most robust predictors of pregnancy-related LBP [7], and likely reflects an underlying predisposition, whether biomechanical, postural, or related to core muscular strength, that persists across pregnancies.

These findings point toward specific, actionable strategies for antenatal care in this setting. First, given that personal history of low back pain was the strongest associated factor identified, antenatal booking visits could incorporate a brief, targeted screening question on prior LBP history, allowing early identification of women at highest risk for proactive counseling and monitoring, rather than relying on spontaneous symptom reporting. Second, for women identified as at-risk, evidence-based non-pharmacological interventions with demonstrated benefit in pregnancy, such as supervised pregnancy-specific exercise programs, pelvic support belts, and posture and body mechanics education, could be integrated into routine antenatal counseling, consistent with recommendations from international guidelines on musculoskeletal health in pregnancy [15]. Third, given the observed association with reduced activity level and the cultural normalization of pain reported by most affected women in this study, counseling messages should explicitly reframe low back pain as a legitimate, manageable condition rather than an inevitable consequence of pregnancy, to encourage timely reporting and uptake of available interventions. Implementation of these strategies would require minimal additional resources within existing antenatal care visits and could be piloted within the primary healthcare centers included in this study before broader scale-up across Burkina Faso’s health districts.

Our study has several limitations. This was a facility-based study conducted exclusively in public primary healthcare centers, and as such, the reported prevalence may not be generalizable to the general population of pregnant women in Ouagadougou. Women who did not attend antenatal care, those receiving care in private facilities, and women with severe pain-related disability who may have been less able to attend clinic visits were not captured. This selection process may have led to an underestimation of the true prevalence of low back pain in the source population. We had no data regarding psychological status (e.g., depression, anxiety), sleep quality, relaxation level, economic status, occupational workload (including duration of standing), physical exercise habits, weight gain during pregnancy, pain intensity, or the presence of pelvic girdle pain as distinct from low back pain. These unmeasured factors may represent sources of residual confounding and limit the completeness of our associated factor assessment. Newborn birth weight was initially collected via telephone follow-up but was ultimately excluded from all analyses due to substantial missingness (available for only 296 of 1,159 participants, 74.46%) and its temporal position after the antenatal low back pain assessment, which precluded meaningful interpretation. However, our study is one of the largest studies in Africa. Additionally, low back pain was assessed as a period prevalence since the onset of pregnancy rather than a point prevalence with a defined recall window, and pain intensity was not measured. This may limit direct comparability with studies using different case definitions and may have contributed to the lower prevalence observed relative to other reports. Furthermore, the number of women approached and the number who declined participation were not recorded during data collection, precluding calculation of a response rate. While recruitment aimed to be consecutive among women presenting for antenatal care during the study period, this lack of documentation does not allow formal assessment of non-participation bias. As with all cross-sectional studies, temporal sequence between exposures and outcome cannot be established, which is particularly relevant for the association observed between activity level and low back pain, where reverse causation cannot be excluded. Regarding the assessment of impact, our study captured only limited dimensions of disability, namely the Pregnancy Mobility Index, self-perceived significance of pain, and activity level. Other important dimensions of impact, such as sleep disturbance, work absenteeism, mental health, healthcare utilization, and analgesic use, were not assessed and should be considered in future studies. Additionally, pelvic girdle pain (PGP) was not formally differentiated from low back pain in the questionnaire; only women with a documented history of pelvic trauma or surgery were excluded, which does not preclude symptomatic overlap between PGP and LBP in the reported outcome. Although the final model showed acceptable discrimination (AUC = 0.699) and explained a moderate proportion of variability (pseudo-R^2^ = 0.152), unmeasured factors such as psychological distress, occupational workload, or pain intensity likely contribute additional explanatory power beyond the variables captured in this study. Missing data were minimal for most sociodemographic and clinical variables analyzed (0% missing after recoding non-selected checkbox items as absent, per the skip-logic structure of the electronic questionnaire). As previously noted, newborn birth weight was available for only 296 of 1,159 participants (74.46% missing), and the Pregnancy Mobility Index was, by design, administered only to the 251 women reporting low back pain.

## Conclusion

Low back pain is a common but frequently under-recognized musculoskeletal condition among pregnant women attending antenatal care in Ouagadougou, affecting roughly one in five participants. Despite its documented impact on daily activity, most affected women perceived their condition as insignificant, highlighting a gap between objective functional limitation and subjective symptom recognition in this setting. Urban residence, reduced activity level, and personal history of low back pain emerged as the strongest associated factors, with prior history showing a particularly robust association. These findings support the integration of routine, proactive musculoskeletal screening into antenatal care protocols, rather than relying on spontaneous patient reporting, particularly for women with a known history of back pain. Further prospective and longitudinal research incorporating psychological, occupational, and biomechanical factors is warranted to better characterize the determinants and trajectory of pregnancy-related low back pain in sub-Saharan African settings, and to inform the development of context-appropriate preventive and management strategies.

## Supporting information

S1 Checklist STROBE checklist for cross-sectional studies. (PDF)

S2 Dataseat De-identified dataset of the study. (CSV)

## Abbreviations

LBP: Low back pain
PMI: Pregnancy mobility index
