# Supplementary material for: Prevalence, associated factors, and functional impact of low back pain among pregnant women attending antenatal care in Ouagadougou, Burkina Faso: A cross-sectional study
Source: PLoS One. 2026 Oct 23;21(10):e0360908. doi: 10.1371/journal.pone.0360908 (PMC13693925; doi:10.1371/journal.pone.0360908)
Supplement: Supplementary file 1 [file pone.0360908.s001.pdf]

STROBE Checklist

Item	Present	Page
1.a Title and abstract	☒	1
1.b Summary and objective information on what was done and found in the abstract	☒	2
2.a Scientific background of the study	☒	3
2.b Rationale of the study	☒	3
3.a Specific objectives of the study	☒	3
3.b A priori hypotheses of the study	☒	3
3.c Research question	☒	3
4. Study design	☒	4
5. Setting, locations, relevant dates, periods of recruitment, exposure, follow-up and data collection	☒	4
6. Description of the population adapted to the type of study	☒	4
7. Variables: clear description of outcome criteria, exposure, prediction factors, potential confounding factors, etc.	☒	5
8. Indication of data sources and evaluation methods for each variable of interest	☒	5
9. Description of all measures taken to avoid potential sources of bias	☒	6
10. Explanation of the number of subjects to include	☒	6
11. Treatment of quantitative variables and justification	☒	6
12.a Description of all statistical analyses	☒	6
12.b Description of methods used	☒	6
12.c Description of missing data treatment	☒	7

Item	Present	Page
12.d Description of specific data treatment by study type (lost to follow-up, case-control matching, sampling)	☒	7
12.e Description of sensitivity analyses	☐	
13. Description of the population at each stage of the study, number of individuals, reasons for non-participation, presence of a flow diagram	☐	
14.a Description of the characteristics of the studied population	☒	7
14.b Description of the number of subjects and missing data for each variable	☒	7
14.c For cohort studies, summary of the follow-up period	☐	
15. Description of obtained data (adapted to study type) (e.g., number of events, number of exposed or unexposed subjects)	☒	8
16.a Description of main results: unadjusted, adjusted estimates, presence of confidence interval	☒	9
17. Description of other analyses performed: subgroup analysis, sensitivity analysis	☒	9
18. Summary of main results	☒	10
19. Discussion of study limitations	☒	13
20. General interpretation of results taking into account objectives, study limitations, etc.	☒	12
21. Discussion of the generalizability of study results	☒	11
22. Source of funding	☐	
