# Supplementary material for: Prevalence, associated factors, and functional impact of low back pain among pregnant women attending antenatal care in Ouagadougou, Burkina Faso: A cross-sectional study
Source: PLoS One. 2026 Oct 23;21(10):e0360908. doi: 10.1371/journal.pone.0360908 (PMC13693925; doi:10.1371/journal.pone.0360908)
Supplement: Supplementary file 2 [file pone.0360908.s005.docx]

**Response Reviewser 4**

We sincerely thank Reviewer #4 for their thorough engagement with our manuscript across both rounds of review, and for confirming that all previous comments have been adequately addressed. We greatly appreciate the time and expertise invested in helping us strengthen this work.
