# Supplementary material for: Prevalence, associated factors, and functional impact of low back pain among pregnant women attending antenatal care in Ouagadougou, Burkina Faso: A cross-sectional study
Source: PLoS One. 2026 Oct 23;21(10):e0360908. doi: 10.1371/journal.pone.0360908 (PMC13693925; doi:10.1371/journal.pone.0360908)
Supplement: Peer review history

# Peer review: Decision Letter 0

**DOI:** 10.1371/journal.pone.0360908.r001

**Authors:** Clement Yaro

29 Jun 2026

Dear Dr. Ouédraogo,

Thank you for submitting your manuscript to PLOS ONE. After careful consideration, we feel that it has merit but does not fully meet PLOS ONE’s publication criteria as it currently stands. Therefore, we invite you to submit a revised version of the manuscript that addresses the points raised during the review process.

Please submit your revised manuscript by Aug 13 2026 11:59PM. If you will need more time than this to complete your revisions, please reply to this message or contact the journal office at plosone@plos.org. When you're ready to submit your revision, log on to https://www.editorialmanager.com/pone/ and select the 'Submissions Needing Revision' folder to locate your manuscript file.

- A letter that responds to each point raised by the academic editor and reviewer(s). You should upload this letter as a separate file labeled 'Response to Reviewers'.
- A marked-up copy of your manuscript that highlights changes made to the original version. You should upload this as a separate file labeled 'Revised Manuscript with Track Changes'.
- An unmarked version of your revised paper without tracked changes. You should upload this as a separate file labeled 'Manuscript'.

If you would like to make changes to your financial disclosure, please include your updated statement in your cover letter. Guidelines for resubmitting your figure files are available below the reviewer comments at the end of this letter.

If applicable, we recommend that you deposit your laboratory protocols in protocols.io to enhance the reproducibility of your results. Protocols.io assigns your protocol its own identifier (DOI) so that it can be cited independently in the future. For instructions see: https://journals.plos.org/plosone/s/submission-guidelines#loc-laboratory-protocols. Additionally, PLOS ONE offers an option for publishing peer-reviewed Lab Protocol articles, which describe protocols hosted on protocols.io. Read more information on sharing protocols at https://plos.org/protocols?utm_medium=editorial-email&utm_source=authorletters&utm_campaign=protocols.

As the corresponding author, your ORCID iD is verified in the submission system and will appear in the published article. PLOS supports the use of ORCID, and we encourage all coauthors to register for an ORCID iD and use it as well. Please encourage your coauthors to verify their ORCID iD within the submission system before final acceptance, as unverified ORCID iDs will not appear in the published article. *Only*  the individual author can complete the verification step; PLOS staff *cannot*  verify ORCID iDs on behalf of authors.

We look forward to receiving your revised manuscript.

Kind regards,

Clement Ameh Yaro, Ph.D

Academic Editor

PLOS One

Journal Requirements:

When submitting your revision, we need you to address these additional requirements.

1. Please ensure that your manuscript meets PLOS ONE’s style requirements, including those for file naming. The PLOS ONE style templates can be found at

https://journals.plos.org/plosone/s/file?id=wjVg/PLOSOne_formatting_sample_main_body.pdf and https://journals.plos.org/plosone/s/file?id=ba62/PLOSOne_formatting_sample_title_authors_affiliations.pdf

2. We suggest you thoroughly copyedit your manuscript for language usage, spelling, and grammar. If you do not know anyone who can help you do this, you may wish to consider employing a professional scientific editing service.

The American Journal Experts (AJE) (https://www.aje.com/) is one such service that has extensive experience helping authors meet PLOS guidelines and can provide language editing, translation, manuscript formatting, and figure formatting to ensure your manuscript meets our submission guidelines. Please note that having the manuscript copyedited by AJE or any other editing services does not guarantee selection for peer review or acceptance for publication.

Upon resubmission, please provide the following:

The name of the colleague or the details of the professional service that edited your manuscript

A copy of your manuscript showing your changes by either highlighting them or using track changes (uploaded as a *supporting information* file)

A clean copy of the edited manuscript (uploaded as the new *manuscript* file).

3. Your ethics statement should only appear in the Methods section of your manuscript. If your ethics statement is written in any section besides the Methods, please delete it from any other section.

4. We note that your Data Availability Statement is currently as follows: All relevant data are within the manuscript and its Supporting Information files.

Please confirm at this time whether or not your submission contains all raw data required to replicate the results of your study. Authors must share the “minimal data set” for their submission. PLOS defines the minimal data set to consist of the data required to replicate all study findings reported in the article, as well as related metadata and methods (https://journals.plos.org/plosone/s/data-availability#loc-minimal-data-set-definition).

For example, authors should submit the following data:

- The values behind the means, standard deviations and other measures reported;

- The values used to build graphs;

- The points extracted from images for analysis.

Authors do not need to submit their entire data set if only a portion of the data was used in the reported study.

If your submission does not contain these data, please either upload them as Supporting Information files or deposit them to a stable, public repository and provide us with the relevant URLs, DOIs, or accession numbers. For a list of recommended repositories, please see https://journals.plos.org/plosone/s/recommended-repositories.

If there are ethical or legal restrictions on sharing a de-identified data set, please explain them in detail (e.g., data contain potentially sensitive information, data are owned by a third-party organization, etc.) and who has imposed them (e.g., an ethics committee). Please also provide contact information for a data access committee, ethics committee, or other institutional body to which data requests may be sent. If data are owned by a third party, please indicate how others may request data access.

5. If the reviewer comments include a recommendation to cite specific previously published works, please review and evaluate these publications to determine whether they are relevant and should be cited. There is no requirement to cite these works unless the editor has indicated otherwise.

[Note: HTML markup is below. Please do not edit.]

Reviewers' comments:

Reviewer’s Responses to Questions

**Comments to the Author**

1. Is the manuscript technically sound, and do the data support the conclusions?

Reviewer #1: Yes

Reviewer #2: Partly

Reviewer #3: Partly

Reviewer #4: No

**********

2. Has the statistical analysis been performed appropriately and rigorously?

Reviewer #1: Yes

Reviewer #2: No

Reviewer #3: No

Reviewer #4: No

**********

3. Have the authors made all data underlying the findings in their manuscript fully available?

The PLOS Data policy

Reviewer #1: Yes

Reviewer #2: Yes

Reviewer #3: No

Reviewer #4: Yes

**********

4. Is the manuscript presented in an intelligible fashion and written in standard English?

Reviewer #1: Yes

Reviewer #2: No

Reviewer #3: Yes

Reviewer #4: No

**********

Reviewer #1: Reviewer Report

Manuscript: Low back pain during pregnancy: Prevalence, risk factors and impact among pregnant women in Ouagadougou, Burkina Faso

Recommendation: Major Revision

This manuscript investigates the prevalence, associated factors, and impact of low back pain (LBP) among pregnant women attending antenatal care clinics in Ouagadougou, Burkina Faso. The topic is clinically relevant because pregnancy-related LBP is a major contributor to maternal discomfort, reduced mobility, and impaired quality of life. The study’s strengths include a large sample size (n = 1,159), multicenter recruitment from 14 primary healthcare facilities, and the use of a validated mobility assessment instrument (PMI). However, several methodological, analytical, and interpretative concerns limit confidence in the conclusions. Substantial revision is required before the manuscript can be considered for publication.

---

Major Comments

1. The reported prevalence is substantially lower than most published literature and requires deeper explanation

The prevalence of LBP was reported as 21.8%, which is considerably lower than estimates from recent systematic reviews (approximately 40–50%) and lower than many African studies cited by the authors.

However, the manuscript does not adequately explore why such a discrepancy exists.

Possible explanations include:

Differences in LBP definition.

Point prevalence versus period prevalence.

Underreporting due to cultural normalization of pain.

Facility-based sampling.

Recall period used.

Inclusion of women across all trimesters.

Recommendation

Provide a more detailed methodological comparison with previous studies and discuss why prevalence in Burkina Faso may be considerably lower than global estimates.

---

2. Case definition of low back pain is insufficiently operationalized

The manuscript defines LBP anatomically but does not specify:

Recall period (current pain, pain during pregnancy, pain in the last week, last month, etc.).

Minimum duration.

Minimum pain intensity.

Whether recurrent and chronic pain were differentiated.

Why this matters

Prevalence estimates vary greatly depending on the operational definition.

Recommendation

Clearly specify:

Recall timeframe.

Diagnostic criteria.

Whether self-report alone was used.

Whether pain intensity was measured.

---

3. Sampling strategy may introduce selection bias

Participants were recruited exclusively from antenatal care attendees in selected high-volume health centers.

This raises concerns because:

Women not attending ANC were excluded.

Women attending private facilities were excluded.

Women with severe disability may have been less likely to attend clinics.

Recommendation

Discuss selection bias more comprehensively.

The statement that this study reflects all pregnant women in Ouagadougou is not justified.

---

4. Consecutive recruitment may compromise representativeness

The authors recruited all women presenting during the study period.

However:

No information is provided on clinic attendance patterns.

No sampling interval was used.

No response rate is reported.

Recommendation

Report:

Number approached.

Number refused.

Response rate.

Reasons for non-participation.

This is required for evaluating potential recruitment bias.

---

5. The association between gestational age and uterine height suggests possible multicollinearity

The final model includes both:

Gestational age.

Uterine height.

These variables are biologically highly correlated.

Yet:

Gestational age appears protective (aOR 0.96).

Uterine height appears harmful (aOR 1.09).

This result is counterintuitive because uterine height generally increases with gestational age.

Recommendation

Authors should:

Assess multicollinearity formally.

Report VIF values.

Consider excluding one of the two variables.

Explain the biological plausibility of opposite effects.

This is one of the most important methodological concerns in the paper.

---

6. Interpretation of activity level is problematic

The final model indicates:

> Low activity level reduced the odds of LBP (aOR = 0.72).

However, activity level was measured simultaneously with LBP.

Women experiencing pain may reduce activity because of pain.

Thus reverse causation is highly likely.

Recommendation

The discussion should acknowledge that:

Reduced activity may be a consequence rather than a cause of LBP.

The cross-sectional design prevents temporal inference.

The current interpretation overstates causality.

---

7. Educational level findings are unstable and difficult to interpret

Only 24 women belonged to the “French-Arab school/professional school” category.

Yet this category produced:

> aOR = 3.98 (95% CI 1.41–11.22)

The wide confidence interval suggests substantial instability.

Recommendation

Consider:

Combining educational categories.

Providing category frequencies among cases and controls.

Interpreting this finding cautiously.

Currently, the conclusion emphasizes education despite weak precision.

---

8. Important known risk factors were not measured

Several established predictors of pregnancy-related LBP were absent:

Psychological distress.

Depression/anxiety.

Sleep quality.

Occupational workload.

Duration of standing.

Physical exercise.

Previous pregnancy-related LBP.

Pain intensity.

Pelvic girdle pain.

The authors acknowledge some omissions but not all.

Recommendation

Expand the limitations section and discuss potential residual confounding.

---

9. History of low back pain should not disappear from the final model without explanation

Univariate analysis showed a very strong association:

71.9% among women with prior LBP versus 15.8% without prior LBP.

However, in multivariable analysis:

OR = 1.76

p = 0.051

This near-significant result is not discussed.

Recommendation

Discuss why this well-established predictor lost significance after adjustment.

---

10. The impact assessment is superficial

One of the study objectives was to assess impact.

However impact assessment was limited to:

PMI score.

Self-perceived significance.

Activity level.

No assessment was performed regarding:

Sleep disturbances.

Work absenteeism.

Mental health.

Healthcare utilization.

Analgesic use.

Recommendation

The manuscript should acknowledge that the impact assessment captures only limited dimensions of disability.

---

Statistical Comments

11. The sample size justification is inadequate

The minimum calculated sample size was 380.

The authors enrolled 1,159 participants because they wanted “more power.”

Concern

This is not a sufficient methodological justification.

Recommendation

Provide:

Expected effect size.

Precision target.

Design effect if clustering existed.

Rationale for tripling the calculated sample.

---

12. Use of p ≤ 20% for model selection needs justification

Variables with p ≤ 0.20 entered multivariable analysis.

Recommendation

Clarify:

Whether backward elimination was used.

Whether theoretical variables were retained regardless of significance.

How the final model was selected.

---

13. No regression diagnostics are reported

The manuscript does not report:

Hosmer–Lemeshow test.

ROC curve.

Model discrimination.

Goodness-of-fit statistics.

Multicollinearity diagnostics.

Recommendation

Provide comprehensive model diagnostics.

---

14. Continuous variables should be checked for linearity

Variables modeled continuously include:

Gestational age.

BMI.

Uterine height.

Weight.

Gravidity.

Recommendation

Assess linearity in the logit.

If violated, use categorized or spline-based models.

---

15. Birth weight analysis is methodologically problematic

Birth weight was available for only 296 of 1,159 participants.

Moreover:

Birth weight occurs after delivery.

LBP occurred during pregnancy.

Concern

Birth weight cannot reasonably be considered a predictor of antenatal LBP.

Recommendation

Remove birth weight from risk factor analyses or treat it as a secondary exploratory outcome.

---

Results Reporting Comments

16. Tables contain several inconsistencies

In Table 5:

Marital status lists:

Single

Single

Married

Widow

One of these categories appears to be cohabiting/concubinage.

Recommendation

Correct all table labeling errors.

---

17. Gestational age results appear contradictory

Table 5 suggests:

Highest prevalence occurs in the second trimester.

Yet multivariable analysis indicates:

Increasing gestational age reduces risk.

Recommendation

Provide graphical analysis of LBP prevalence across gestational age.

The relationship may be nonlinear.

---

18. PMI interpretation is unclear

The mean PMI was reported as:

13.31 ± 9.40

However:

Possible score range is not provided.

Thresholds for mild, moderate, and severe impairment are absent.

Recommendation

Explain clinical interpretation of PMI values.

---

Discussion Comments

19. The discussion overstates causal interpretations

Statements such as:

> educational level and uterine height were identified as significant risk factors

are too strong for a cross-sectional study.

Recommendation

Use:

> “associated factors”

instead of:

> “risk factors”

throughout the manuscript.

---

20. Biological explanation regarding lumbar hyperlordosis is speculative

The discussion states:

> lumbar hyperlordosis observed in black African women is accentuated during pregnancy

as an explanation for uterine height findings.

However:

Hyperlordosis was not measured.

Spinal curvature was not assessed.

Recommendation

Present this only as a hypothesis and provide supporting citations.

---

21. Cultural normalization of pain deserves deeper discussion

An interesting finding is that:

71.7% considered their pain insignificant despite activity limitations.

This could reflect:

Cultural beliefs.

Expectations regarding pregnancy.

Limited healthcare-seeking behavior.

Recommendation

Expand this discussion because it may represent the manuscript’s most novel contribution.

---

Reporting and Presentation Comments

22. Data availability statement is inconsistent

The submission form states:

> All relevant data are within the manuscript and Supporting Information files.

However, the manuscript states:

> datasets are available from the corresponding author upon reasonable request.

Recommendation

Ensure consistency with PLOS ONE data-sharing requirements.

---

23. Ethical statement contains problematic wording

The manuscript states:

> informed consent was obtained from each included patient or their relatives.

Pregnant women are competent adults.

Recommendation

Explain under what circumstances consent was obtained from relatives.

This wording raises ethical concerns.

---

24. English language requires substantial editing

Examples include:

“heath districts” instead of “health districts.”

Multiple punctuation inconsistencies.

Several grammatical errors.

Repeated wording.

Recommendation

Professional language editing is recommended.

---

25. Title should reflect study design

Suggested revision:

> “Prevalence, associated factors, and functional impact of low back pain among pregnant women attending antenatal care in Ouagadougou, Burkina Faso: A cross-sectional study”

This would better reflect the study design and setting.

Minor Comments

1. Report response rate.

2. Clarify whether pelvic girdle pain was excluded.

3. Define PMI scoring range.

4. Report pain intensity measures if collected.

5. State how missing data were handled.

6. Report missingness for all variables.

7. Include STROBE checklist.

8. Clarify whether investigators were trained and standardized.

9. Provide a flow diagram of participant recruitment.

10. Standardize terminology ("pregnancy age", "gestational age", and "weeks of amenorrhea").

Overall Recommendation

Major Revision

The study addresses an important and under-researched topic in sub-Saharan Africa and benefits from a large sample size. However, important concerns regarding outcome definition, sampling methodology, model construction, interpretation of associations, and reporting quality currently limit the robustness of the conclusions. Addressing these issues would substantially strengthen the manuscript and improve its contribution to the literature on pregnancy-related low back pain.

Reviewer #2: -Title page :

oThe affiliations should be revised. I would consider “Joseph Ki-Zerbo University” and “University Teaching Hospital.”

o

-Abstract

oInconsistency: The objectives mention risk factors, while in the methods section, the last sentence is about factors associated with LBP

-Methods

oThe study setting does not provide a background relevant to LBP and the study population

oThere are more than 13 health districts in Burkina Faso

oThere has been no tangible explanation of why and how the sample size of 380 became 1159

oThe variables are insufficiently described. The dependent variable has no reference to the categorization: LBP is considered as reported by the woman?

oThe phrasing in the data analysis section is incorrect and should be improved

-Data analysis section report on data collection, tools, and measurement in the last paragraph. These were supposed to be reported and referenced in the variables or measurements section

-The ethical considerations section should go beyond the approvals and authorizations to detail how ethical principles were applied in this study. For instance, in the results section, we note that there are fewer than 15 women, who are minors. How were their assent and informed consent processed?

-Results

oTable 1: it is good practice to keep one decimal place for the univariable analysis percentages

oTable 1: why is age excluded?

oTable 1: remove environment

oTable 1: starting socioprofessional groups by “others” is not common. Same for education

oTable 1: marital status categories include "widow" with only one woman, which is not appropriate. I would suggest two categories: in couple/not in couple

oTable 1: what is the meaning of “out-of-school” in this context?

oTable 1: The authors have two instances of Other and only one footnote of Other. Which one does it refer to?

oThe authors should restructure the reporting. It appears in the results section that they collected that from women who delivered in a subsample. This was not presented in the methods section, and the rationale is unknown.

oTable 2: a lot of inappropriate wordings: e.g., fundal height 1st trimester

oPrevalence: a section of only one sentence

o3.3 Socioprofessional impact: this wording is not appropriate: in Epidemiology, the word impact refers to a different concept. To me, this section reports frequencies related to the mobility and activity of the women. There is no measure of impact in this work.

oTable 3 reports on 251 women with an explanation of why?

oIn the univariate section, the authors should report OR and their IC95%

oThe authors should rather consider the wording “multivariable” instead of “Multivariate” analysis.

oThe authors should be consistent. The objectives were to identify risk factors. But in the results, they report both protective and risk factors.

oTable 6: the multivariable analysis is invalid for 2 reasons: 1) the fact that the authors included the variable “activity level”, which was reported for only 251 women, excludes all the other women. 2) with 21% prevalence of LBP, I would rather suggest a modified Poisson regression to compute prevalence ratios instead of OR.

Reviewer #3: Major Comments

1. Methodological and statistical concerns

•The study has a robust sample size (n=1159) that significantly exceeds the minimum required sample size (n=380), thus providing strong statistical power to the analysis. However, some conclusions drawn from the multivariate analysis have significant epidemiological limitations or unexpected formulations- for instance the protective effect of advanced gestational age when the crude prevalence is highest in the third trimester- that require major corrections.

•Analysis and processing of data : Please provide detail on the statistical models used for analyses:

oUnivariate analysis, Table 4, which statistical model was used to assess association as the independent variables are quantitative. Please provide detail in the “Analysis and processing of data” section.

oUnivariate analysis, Table 5, which statistical model was used to assess association as the independent variables are categorial. Please provide detail in the “Analysis and processing of data” section.

oOmission of key predictors: In Table 5, a history of low back pain (Personal history of LBP) is one of the strongest univariately associated factors (71.90% vs 15.80%, p<0.001). In Table 6, it remains borderline significant in the initial model (aOR=1.760,p=0.051). Omitting this major clinical confounder from the final model without an explicit epidemiological justification is a major flaw that creates residual confounding.

oCollinearity artefact (Table 6): The final model reports advanced gestational age as a protective factor (aOR=0.96, p=0.006), which directly contradicts both common clinical knowledge and the authors' own univariate results (where LBP is significantly more prevalent in the 2nd and 3rd trimesters: 91 and 138 cases respectively, compared to only 22 cases in the 1st trimester). This is a classic sign of multicollinearity because gestational age and uterine height (aOR=1.09, p<0.001) are highly correlated. The authors must compute Variance Inflation Factors (VIF) and adjust their modeling strategy accordingly.

2.Discussion

In the discussion, the results are no longer reported as they have already been presented in the "Results" section; the significance of the results must be explained. Furthermore, you have simply listed pain prevalence rates from other studies without providing a comparison. Please review the entire discussion and include:

•the meaning of your results (low, medium, or high)

•the comparison with other studies (similar, lower, or higher)

•a plausible explanation for the result in question

•and the significance of the findings in terms of clinical or public health implications

Interpretation of "Low activity level" (Table 6): The authors state that a "low activity level" is a protective factor (aOR=0.72, p=0.038). However, in a cross-sectional design, it is highly probable that women with low back pain reduced their activity level because of the pain (reverse causality). This temporal ambiguity must be heavily emphasized in the Discussion as a core limitation of the cross-sectional design.

3. Inconsistencies in the abstract versus main text

In the Abstract (Results), it is written: "Multivariate analysis revealed that vocational training without prior schooling (aOR: 3.98...) was a risk factor". However, looking at Table 1 and Table 5, this specific category is labeled as "Other (french-arab school / vocational school)" or "French-arabic school/professional". The phrase "without prior schooling" is not supported by the data tables and must be harmonized.

Minor Comments

1. Data Availability Policy Contradiction

Authors must explain clearly the reasons for restricting access to the data.

2. Table formatting and presentation

•Table 1 and Table 5 categories: Please standardize category names between tables. For instance, Marital status lists "Concubinage" in Table 1 but Table 5 lists "Single" twice in the rows. This is a typographical error that needs correction (the first row likely corresponds to Concubinage).

•Table 2 and Table 6 labels: For Uterine height and BMI, the Mean ± SD values are strictly identical in Table 2 (25.76±4.65). Please double-check if this is a copy-paste error.

•The study period: For the data collection period “01/02/2024 to 30/05/2024” did not matched the period sated in Tables “February 19 to May 03, 2024”, please clarify.

•Foot note of tables 1 and 6: Please clearly indicate what is referenced by the asterisk “*”.

•Please comment the results of the Tables: for instance “The mean birth weight of the baby was 3134.41 g ± 645.71 g” in not related to the Table 2.

•In tables, I suggest to use “range” instead of “extremes” and replace “and” by “-“ between numbers. It will give more clarity to the tables.

•Tables 4 and 5, please comment separately the results of the tables just before them.

3. Formating, language and terminology

•Please replace the French medical expression "weeks of amenorrhea" (used in Table 2 and Table 6) with standard international English terminology: "weeks of gestation" or "gestational age (weeks)".

•Page 8, Discussion: "The lumbar hyperlordosis observed in black African women is accentuated during pregnancy...". This statement requires a solid, peer-reviewed anthropological or medical reference, as making generalized anatomical assertions based on race without data or literature support weakens the scientific text

•Keywords: Please add the following to the list of keywords “low back pain” instead of ”low back”, “pregnancy”, and “gestational age”

•Corresponding author: according to the PLOS ONE guide lines, Corresponding author must be marked by (*) and his address be presented as follow E-mail: testperson@university.ed (MT)

Please refer to https://journals.plos.org/plosone/s/file?id=ba62/PLOSOne_formatting_sample_title_authors_affiliations.pdf for authors presentation

•Line number: lines should be numbered from the title to the end of the document

•Affiliation 2, please move “Ouagadougou” to place before “Burkina Faso”

•To format the main body of the article, please refer to https://journals.plos.org/plosone/s/file?id=wjVg/PLOSOne_formatting_sample_main_body.pdf (the head title should not be numbered, please harmonize the police in the main body of the article)

4. Conclusion

In the conclusion, the study results are no longer reported in numerical terms. Instead, the major findings should be summarized (qualifying them) and the conclusions should be presented directly, outlining the interventions they call for and the future outlook.

5. Authors’ contributions

For this section please refer to https://journals.plos.org/plosone/s/file?id=ba62/PLOSOne_formatting_sample_title_authors_affiliations.pdf

Reviewer #4: This manuscript investigates the prevalence, associated factors, and socio-occupational impact of low back pain among pregnant women attending antenatal care clinics in Ouagadougou, Burkina Faso. The study addresses an important public health issue, particularly given the limited evidence from sub-Saharan Africa, and benefits from a relatively large sample size.

However, despite these strengths, the manuscript does not currently meet the reporting standards expected for observational studies (according to the STROBE Statement for example). Several important methodological and reporting deficiencies substantially limit the assessment of the internal validity of the study and the credibility of the reported findings. These concerns mainly relate to participant selection, missing data, statistical modelling, reporting of potential sources of bias, and interpretation of the results.

Major concerns

1.Participant selection, sample size, and ethical consideration

The recruitment process is insufficiently described to assess representativeness and potential selection bias. The manuscript should clearly report the number of eligible women, those approached, excluded (with reasons), and refusals, and specify whether recruitment was consecutive or convenience based. In addition, the substantial discrepancy between the calculated sample size (n = 380) and the final sample (n = 1,159) requires explicit justification. It should be clarified whether this reflects a planned exhaustive recruitment over the study period or a post hoc extension of the original sample size calculation. Furthermore, the authors should confirm whether this increase in sample size was covered under the original ethical approval (or an approved amendment), and whether identical informed consent procedures were applied to all participants.

2.Missing data management

Missing data represent one of the major weaknesses of the current manuscript. For instance, birth weight is available for only 296 out of 1 159 participants (around 25%), yet this variable is used in statistical analyses without detailed explanation. The manuscript does not clarify. Reasons for missing birth weight data (e.g. not yet delivered at the time of interview, lack of measurement, missing records).

3.Terminology and causal interpretation

The manuscript frequently uses causal terminology (e.g., “risk factors”) despite the cross-sectional design. This is not appropriate given the lack of temporality. The authors should replace causal language with appropriate terms such as “associated factors” or “correlates” throughout the manuscript. The Discussion and Conclusion sections should also be revised to avoid causal inference and to explicitly acknowledge the limitations of cross-sectional data in establishing causality.

4.Important discrepancies are observed between the descriptive analyses and the multivariable regression results. For example, crude results suggest that physically inactive women have a higher prevalence of low back pain, whereas the adjusted model indicates that low physical activity has a protective effect (aOR < 1). Similarly, gestational age appears positively associated with low back pain in the descriptive analyses but becomes protective after adjustment. Such reversals may be explained by:

•Confounding between physical activity, occupational demands, and other sociodemographic variables.

•Multicollinearity between gestational age, parity, and other pregnancy-related variables.

•Model instability due to variable selection based solely on p-values.

**********

what does this mean?). If published, this will include your full peer review and any attached files.

If you choose “no”, your identity will remain anonymous but your review may still be made public.

**Do you want your identity to be public for this peer review?**  For information about this choice, including consent withdrawal, please see our Privacy Policy

Reviewer #1: **Yes:**  Habtamu Fekadu Gemede

Reviewer #2: **Yes:**  Adama Baguiya

Reviewer #3: No

Reviewer #4: No

**********

[NOTE: If reviewer comments were submitted as an attachment file, they will be attached to this email and accessible via the submission site. Please log into your account, locate the manuscript record, and check for the action link "View Attachments". If this link does not appear, there are no attachment files.]

To ensure your figures meet our technical requirements, please review our figure guidelines: https://journals.plos.org/plosone/s/figures

You may also use PLOS’s free figure tool, NAAS, to help you prepare publication quality figures: https://journals.plos.org/plosone/s/figures#loc-tools-for-figure-preparation.

NAAS will assess whether your figures meet our technical requirements by comparing each figure against our figure specifications.

# Author comment: Author response to Decision Letter 1

**DOI:** 10.1371/journal.pone.0360908.r002

13 Jul 2026

Supplementary Information

# Peer review: Decision Letter 1

**DOI:** 10.1371/journal.pone.0360908.r003

**Authors:** Clement Yaro

28 Aug 2026

Dear Dr. Ouédraogo,

Thank you for submitting your manuscript to PLOS One. After careful consideration, we feel that it has merit but does not fully meet PLOS One’s publication criteria as it currently stands. Therefore, we invite you to submit a revised version of the manuscript that addresses the points raised during the review process.

Please submit your revised manuscript by Oct 12 2026 11:59PM. If you will need more time than this to complete your revisions, please reply to this message or contact the journal office at plosone@plos.org. When you're ready to submit your revision, log on to https://www.editorialmanager.com/pone/ and select the 'Submissions Needing Revision' folder to locate your manuscript file.

- A letter that responds to each point raised by the academic editor and reviewer(s). You should upload this letter as a separate file labeled 'Response to Reviewers'.
- A marked-up copy of your manuscript that highlights changes made to the original version. You should upload this as a separate file labeled 'Revised Manuscript with Track Changes'.
- An unmarked version of your revised paper without tracked changes. You should upload this as a separate file labeled 'Manuscript'.

If you would like to make changes to your financial disclosure, please include your updated statement in your cover letter. Guidelines for resubmitting your figure files are available below the reviewer comments at the end of this letter.

If applicable, we recommend that you deposit your laboratory protocols in protocols.io to enhance the reproducibility of your results. Protocols.io assigns your protocol its own identifier (DOI) so that it can be cited independently in the future. For instructions see: https://journals.plos.org/plosone/s/submission-guidelines#loc-laboratory-protocols. Additionally, PLOS One offers an option for publishing peer-reviewed Lab Protocol articles, which describe protocols hosted on protocols.io. Read more information on sharing protocols at https://plos.org/protocols?utm_medium=editorial-email&utm_source=authorletters&utm_campaign=protocols.

As the corresponding author, your ORCID iD is verified in the submission system and will appear in the published article. PLOS supports the use of ORCID, and we encourage all coauthors to register for an ORCID iD and use it as well. Please encourage your coauthors to verify their ORCID iD within the submission system before final acceptance, as unverified ORCID iDs will not appear in the published article. *Only*  the individual author can complete the verification step; PLOS staff *cannot*  verify ORCID iDs on behalf of authors.

We look forward to receiving your revised manuscript.

Kind regards,

Clement Ameh Yaro, Ph.D

Academic Editor

PLOS One

Journal Requirements:

If the reviewer comments include a recommendation to cite specific previously published works, please review and evaluate these publications to determine whether they are relevant and should be cited. There is no requirement to cite these works unless the editor has indicated otherwise.

[Note: HTML markup is below. Please do not edit.]

Reviewers' comments:

Reviewer’s Responses to Questions

**Comments to the Author**

Reviewer #2: (No Response)

Reviewer #3: All comments have been addressed

Reviewer #4: All comments have been addressed

**********

2. Is the manuscript technically sound, and do the data support the conclusions?

Reviewer #2: Yes

Reviewer #3: Yes

Reviewer #4: Partly

**********

3. Has the statistical analysis been performed appropriately and rigorously?

Reviewer #2: No

Reviewer #3: Yes

Reviewer #4: Yes

**********

4. Have the authors made all data underlying the findings in their manuscript fully available?

The PLOS Data policy

Reviewer #2: Yes

Reviewer #3: Yes

Reviewer #4: Yes

**********

5. Is the manuscript presented in an intelligible fashion and written in standard English?

Reviewer #2: Yes

Reviewer #3: Yes

Reviewer #4: Yes

**********

Reviewer #2: Title: ok

Authors : the first authors has changed. Initially, it was Fulgence, now, it is Mariam. Has this been arranged with the Editor?

Abstract: ok

Introduction

- “Prevalence varies from 3.9--89.9%, with a median of 49.0% from 1982-2002” needs to be rephrased

- Yet, I find the study relevant, but the authors do not clearly explain that in the introduction. What will be the expected usefulness of this study? How will the findings be used to impact clinical practices or policies in favour of pregnant women in the Faso and elsewhere?

Methods

- 20 505 155 and 4,949,953 : use a consistent format throughout

- Research population is not a common wording. Do you mean study population?

- How were the 11 health facilities chosen? Purposively? When did the five districts report the data that you used?

- The sampling is inconsistent and unstructured. First, the study design announced 11 HF, and the sampling says 14 HF. From the look of things, you purposively recruited 1159 women over two weeks in each site. Which is ok for a hospital-based study! Otherwise, from 380 to 1156, no.

- Variables: for the dependent variable, the authors just repeated the definition of the LBP, while we were expecting to see how it was measured on everyone. So, the first and the last paragraph of this section can be combined to explain how it was measured and coded.

- Independent variables are not described: listing the variables is not enough for readers to understand what they are and how they are measured.

- For example, the thorough explanation of PMI score in the data analysis section should be in the variables section to show how data are captured and how the variable is presented in the analysis

- Birthweight was not captured for a subsample. They could not reach all the participants, so it is attrition, not intentional.

- Considering the high prevalence, please test modified Poisson regression to estimate prevalence ratios instead of OR. You might realise that it fits better than Logistic regression

- Ethical considerations include explanations that do not belong there. The last part should be in the response to the authors' comments, not in the manuscript.

Results

- Please report descriptive statistics with only one decimal point in both the main text and tables across the entire manuscript

- In fine, birthweight was not useful in this study and should be removed.

- Nowhere in the results do the authors make reference to the functional impact mentioned in the title.

- The title of Table 3 is not informative: who are the 251?

- Table 5 is partly in French. Please finalize the translation

Discussion

- Please elaborate on the tailored evidence-based strategies suggested in the discussion because that is what we need from such a study

The authors’ contributions, if accurate, show that Mariam cannot be the first author of this paper.

Please revise the manuscript to correct small language errors or uncommon terminologies like uterine height (fundal height), french in tables, etc.

Reviewer #3: The authors have taken most of my previous comments into account; however, a few points remain to be considered:

- In the results section, the authors described the methods used for the multivariate analysis; this description should have been included in the methods section.

- Table 2 shows a comparison between the two groups: with and without low back pain; please correct the table title to "Comparison" and not "Univariate Association Analysis" because there is no association indicator reported in the table.

- Table 5: the p-values are missing, and the table contains many French words: this needs to be revised.

- The authors repeat the results in the discussion section, even though these results are already presented in the appropriate section. Please remove the results from the discussion.

- The authors have not standardized the expressions "weeks of gestation" and "weeks of amenorrhea"; please use one expression throughout the manuscript.

Reviewer #4: The authors have made considerable efforts to improve the manuscript originally submitted. I have no further comments. Thank you

**********

what does this mean?). If published, this will include your full peer review and any attached files.

If you choose “no”, your identity will remain anonymous but your review may still be made public.

**Do you want your identity to be public for this peer review?**  For information about this choice, including consent withdrawal, please see our Privacy Policy

Reviewer #2: **Yes:**  Adama Baguiya

Reviewer #3: No

Reviewer #4: No

**********

[NOTE: If reviewer comments were submitted as an attachment file, they will be attached to this email and accessible via the submission site. Please log into your account, locate the manuscript record, and check for the action link "View Attachments". If this link does not appear, there are no attachment files.]

To ensure your figures meet our technical requirements, please review our figure guidelines: https://journals.plos.org/plosone/s/figures

You may also use PLOS’s free figure tool, NAAS, to help you prepare publication quality figures: https://journals.plos.org/plosone/s/figures#loc-tools-for-figure-preparation.

NAAS will assess whether your figures meet our technical requirements by comparing each figure against our figure specifications.

# Author comment: Author response to Decision Letter 2

**DOI:** 10.1371/journal.pone.0360908.r004

4 Sep 2026

Dear Editor,

We thank you and the reviewers for their continued engagement with our manuscript "Prevalence, associated factors, and functional impact of low back pain among pregnant women attending antenatal care in Ouagadougou, Burkina Faso: A cross-sectional study" (PONE-D-26-14695R1). We appreciate that Reviewers #3 and #4 found that most or all previous comments had been adequately addressed, and we have carefully considered the remaining points raised by Reviewers #2, #3, and #4 in this round. A detailed, point-by-point response is provided in the accompanying "Response to Reviewers" document.

Journal Requirements

Regarding the recommendation to review reviewer-suggested citations for relevance: we have reviewed all citations suggested across this and the previous round of review and incorporated those judged relevant, as detailed in the Response to Reviewers document.

Note on author order

We would like to reiterate, for the Editor’s record, that the author order was revised from the original submission during the previous round of revision, reflecting an updated agreement among co-authors regarding relative contributions to the manuscript. All authors have approved this change and the author order presented in the current version. We address this point specifically in our response to Reviewer #2 below, who raised a related question.

We believe the manuscript has been further strengthened by this round of revision and thank you again for the opportunity to submit this updated version.

On behalf of all authors,

Dieu-Donné Ouedraogo

Corresponding author

Department of Rheumatology, Health Sciences Training and Research Unit, Joseph Ki-Zerbo University, Ouagadougou, Burkina Faso

Email: dieu-donne.ouedraogo@ujkz.bf

Attachment Submitted filename: Response Reviewser 4.docx

# Peer review: Decision Letter 2

**DOI:** 10.1371/journal.pone.0360908.r005

**Authors:** Clement Yaro

5 Oct 2026

Prevalence, associated factors, and functional impact of low back pain among pregnant women attending antenatal care in Ouagadougou, Burkina Faso: A cross-sectional study

PONE-D-26-14695R2

Dear Dr. Ouédraogo,

We’re pleased to inform you that your manuscript has been judged scientifically suitable for publication and will be formally accepted for publication once it meets all outstanding technical requirements.

Within one week, you’ll receive an e-mail detailing the required amendments. When these have been addressed, you’ll receive a formal acceptance letter and your manuscript will be scheduled for publication.

An invoice will be generated when your article is formally accepted. Please note, if your institution has a publishing partnership with PLOS and your article meets the relevant criteria, all or part of your publication costs will be covered. Please make sure your user information is up-to-date by logging into Editorial Manager at Editorial Manager® and clicking the ‘Update My Information' link at the top of the page. For questions related to billing, please contact billing support.

If your institution or institutions have a press office, please notify them about your upcoming paper to help maximize its impact. If they’ll be preparing press materials, please inform our press team as soon as possible -- no later than 48 hours after receiving the formal acceptance. Your manuscript will remain under strict press embargo until 2 pm Eastern Time on the date of publication. For more information, please contact onepress@plos.org.

Kind regards,

Clement Ameh Yaro, Ph.D

Academic Editor

PLOS One

Additional Editor Comments (optional):

Reviewers' comments:

Reviewer’s Responses to Questions

**Comments to the Author**

Reviewer #3: All comments have been addressed

Reviewer #4: All comments have been addressed

**********

2. Is the manuscript technically sound, and do the data support the conclusions?

Reviewer #3: Yes

Reviewer #4: Yes

**********

3. Has the statistical analysis been performed appropriately and rigorously?

Reviewer #3: Yes

Reviewer #4: Yes

**********

4. Have the authors made all data underlying the findings in their manuscript fully available?

The PLOS Data policy

Reviewer #3: Yes

Reviewer #4: (No Response)

**********

5. Is the manuscript presented in an intelligible fashion and written in standard English?

Reviewer #3: Yes

Reviewer #4: Yes

**********

Reviewer #3: Thank you to the authors.

I wen through the manuscript, and all my first and second comments have been addressed in a proper way.

Reviewer #4: The authors have addressed the comments raised during the review. The revised manuscript has been improved accordingly, and the authors have provided responses to the concerns raised. I am satisfied with the revisions made and have no further major comments.

**********

what does this mean?). If published, this will include your full peer review and any attached files.

If you choose “no”, your identity will remain anonymous but your review may still be made public.

**Do you want your identity to be public for this peer review?**  For information about this choice, including consent withdrawal, please see our Privacy Policy

Reviewer #3: No

Reviewer #4: No

**********

# Editor report: Acceptance letter

**DOI:** 10.1371/journal.pone.0360908.r006

**Authors:** Clement Yaro

PONE-D-26-14695R2

PLOS One

Dear Dr. Ouédraogo,

I'm pleased to inform you that your manuscript has been deemed suitable for publication in PLOS One. Congratulations! Your manuscript is now being handed over to our production team.

At this stage, our production department will prepare your paper for publication. This includes ensuring the following:

* All references, tables, and figures are properly cited

* All relevant supporting information is included in the manuscript submission,

* There are no issues that prevent the paper from being properly typeset

You will receive further instructions from the production team, including instructions on how to review your proof when it is ready. Please keep in mind that we are working through a large volume of accepted articles, so please give us a few days to review your paper and let you know the next and final steps.

Lastly, if your institution or institutions have a press office, please let them know about your upcoming paper now to help maximize its impact. If they'll be preparing press materials, please inform our press team within the next 48 hours. Your manuscript will remain under strict press embargo until 2 pm Eastern Time on the date of publication. For more information, please contact onepress@plos.org.

You will receive an invoice from PLOS for your publication fee after your manuscript has reached the completed accept phase. If you receive an email requesting payment before acceptance or for any other service, this may be a phishing scheme. Learn how to identify phishing emails and protect your accounts at https://explore.plos.org/phishing.

If we can help with anything else, please email us at customercare@plos.org.

Thank you for submitting your work to PLOS One and supporting open access.

Kind regards,

PLOS One Editorial Office Staff

on behalf of

Dr. Clement Ameh Yaro

Academic Editor

PLOS One
